# Mapping clinical interactions in an Australian tertiary hospital emergency department for patients presenting with risk of suicide or self-harm: Network modeling from observational data

**DOI:** 10.1371/journal.pmed.1004241

**Published:** 2024-01-12

**Authors:** Michael H. McCullough, Michael Small, Binu Jayawardena, Sean Hood

**Affiliations:** 1 School of Computing, The Australian National University, Acton, ACT, Australia; 2 Eccles Institute of Neuroscience, John Curtin School of Medical Research, The Australian National University, Acton, ACT, Australia; 3 Complex Systems Group, Department of Mathematics and Statistics, The University of Western Australia, Crawley, WA, Australia; 4 Mineral Resources, Commonwealth Scientific and Industrial Research Organisation, Kensington, WA, Australia; 5 North Metropolitan Health Service, Government of Western Australia, WA, Australia; 6 Division of Psychiatry, UWA Medical School, The University of Western Australia, Crawley, WA, Australia; Massachusetts General Hospital, UNITED STATES

## Abstract

**Background:**

Reliable assessment of suicide and self-harm risk in emergency medicine is critical for effective intervention and treatment of patients affected by mental health disorders. Teams of clinicians face the challenge of rapidly integrating medical history, wide-ranging psychosocial factors, and real-time patient observations to inform diagnosis, treatment, and referral decisions. Patient outcomes therefore depend on the reliable flow of information through networks of clinical staff and information systems. This study aimed to develop a quantitative data-driven research framework for the analysis of information flow in emergency healthcare settings to evaluate clinical practice and operational models for emergency psychiatric care.

**Methods and findings:**

We deployed 2 observers in a tertiary hospital emergency department during 2018 for a total of 118.5 h to record clinical interactions along patient trajectories for presentations with risk of self-harm or suicide (*n* = 272 interactions for *n* = 43 patient trajectories). The study population was reflective of a naturalistic sample of patients presenting to a tertiary emergency department in a metropolitan Australian city. Using the observational data, we constructed a clinical interaction network to model the flow of clinical information at a systems level. Community detection via modularity maximization revealed communities in the network closely aligned with the underlying clinical team structure. The Psychiatric Liaison Nurse (PLN) was identified as the most important agent in the network as quantified by node degree, closeness centrality, and betweenness centrality. Betweenness centrality of the PLN was significantly higher than expected by chance (>95th percentile compared with randomly shuffled networks) and removing the PLN from the network reduced both the global efficiency of the model and the closeness centrality of all doctors. This indicated a potential vulnerability in the system that could negatively impact patient care if the function of the PLN was compromised. We developed an algorithmic strategy to mitigate this risk by targeted strengthening of links between clinical teams using greedy cumulative addition of network edges in the model. Finally, we identified specific interactions along patient trajectories which were most likely to precipitate a psychiatric referral using a machine learning model trained on features from dynamically constructed clinical interaction networks. The main limitation of this study is the use of nonclinical information only (i.e., modeling is based on timing of interactions and agents involved, but not the content or quantity of information transferred during interactions).

**Conclusions:**

This study demonstrates a data-driven research framework, new to the best of our knowledge, to assess and reinforce important information pathways that guide clinical decision processes and provide complementary insights for improving clinical practice and operational models in emergency medicine for patients at risk of suicide or self-harm. Our findings suggest that PLNs can play a crucial role in clinical communication, but overreliance on PLNs may pose risks to reliable information flow. Operational models that utilize PLNs may be made more robust to these risks by improving interdisciplinary communication between doctors. Our research framework could also be applied more broadly to investigate service delivery in different healthcare settings or for other medical specialties, patient groups, or demographics.

## Abstract 2

Michael H McCullough and team present a data-driven research framework to disentangle the complexities of information flow, decision-making, and effective operational practice in psychiatric emergency medicine.

## Author summary

**Why was this study done?:**

> Mental health presentations at emergency departments are unpredictable and require complex responses from emergency healthcare systems.
> Patient outcomes, including risk assessment for self-harm or suicide, depend on reliable flow of information among clinical staff and information systems.
> We developed a data-driven research framework to disentangle the complex flow of information, decision-making and effective operational practice in emergency psychiatric medicine.

**What did the researchers do and find?:**

> A team of observers were deployed to monitor patient interactions with emergency care workers and information systems among emergency presentations deemed to be at risk of self-harm or suicide.
> Data from all presentations were combined to infer a clinical interaction network.
> Techniques from the mathematical field of complexity theory were applied to infer functional interactions between healthcare workers, clinical resources, and information systems.
> In particular, we identified the Psychiatric Liaison Nurse (PLN) as the most important conduit for information flow. Model-based analysis demonstrated that an unexpected absence of the PLN represented a vulnerability of the hospital system.

**What do the findings mean?:**

> PLNs are important for communicating clinical information but care should be taken to avoid overloading staff in this role.
> Improving interdisciplinary communication can enhance the reliability of operational models for emergency psychiatry.
> A data-driven research framework as presented in this study can be used to assess important information pathways in a clinical setting such as emergency psychiatric care and may be applied to other clinical settings.
> The main limitation of this study is the use of nonclinical information only.

## Introduction

Suicide is a major global public health issue causing over 700,000 deaths per year, often with far-reaching impacts on families and communities that can persist well-beyond each individual tragedy [1,2]. In addition, the prevalence of suicide and suicidal ideation creates considerable economic burden for society (estimated at over US$90 billion in the United States of America alone in 2013 [3]) and has been linked to increasing healthcare costs [2].

It has been estimated that as many as 77% of individuals who die by suicide will have made contact with a primary care provider in the year prior to their death [4,5], and up to 10% to 20% will have visited an emergency department (ED) within 1 to 2 months prior [2,6,7]. EDs are an important and often primary point of access for mental health support services [8–10] and therefore provide an opportunity for suicide-risk screening and prevention [2]. However, the population of individuals affected by suicidal behaviors is highly heterogeneous and poses significant challenges for risk assessment and clinical management, especially in emergency settings [11].

Mental health crisis presentations account for 4% to 10% of ED presentations [12] and are growing in number [13–16]. This increases strain on EDs [13] and impacts patient flow because mental health presentations typically take more time to assess and staff often report feeling ill-equipped to care for these patients [12]. Further, EDs are widely understood to be challenging environments for patients affected by mental health issues for reasons including long wait times, noise, lack of privacy, harsh lighting, and negative attitudes of staff [17,18]. These and other factors result in a predominantly negative experience of acute care settings for mental health patients [19]. This is particularly problematic for patients affected by suicidal behaviors because negative experiences of treatment may increase self-harm risk [20].

While clinical management of suicide-risk patients in emergency settings has been prioritized in many national and international suicide prevention strategies [20] existing research into urgent emergency care models for mental health patients is limited, with a lack of evidence-based models in the literature [12]. Notably, patient journeys through EDs for mental health presentations are not well understood [21] and a systematic review of research related to patient experiences in emergency care after self-harm found that there were only a few studies that attempted to describe the care pathway and interactions with medical professionals in detail [20]. Multiple studies also report considerable inconsistencies and discrepancies in the clinical practice guidelines and service delivery models for emergency mental health care in the United States of America, the United Kingdom, Australia, and New Zealand [11,22–26] including a systematic review by Bernert and colleagues [27]. As a result, there have been calls for further research into methods for monitoring and evaluating the implementation of clinical practice guidelines to improve patient experience and treatment outcomes [11,20,28].

We addressed these issues by developing a data-driven research framework to analyze patient trajectories using approaches from network science and machine learning. Specifically, the objective of this investigation was to build systems-level network models that map patient trajectories and clinical information flow for presentations with suspected risk of suicide or self-harm. Using these models, we sought to determine whether aspects of clinical practice or operational structure in the ED could affect the flow of clinical information in ways that could negatively impact patient outcomes, and how potential risks could be mitigated. Furthermore, we explored patient trajectories as dynamic networks to understand the processes of clinical decision-making and referrals as they occur in practice. By quantifying the characteristics of patient trajectories in the ED and the patterns of communication and interaction that drive them, this study aims to provide a research framework to evaluate clinical practice and operational models to guide process improvement and enhance treatment outcomes for patients with suicide or self-harm risk.

## Methods

### Ethics statement

This study was approved as a Quality Activity by the Government of Western Australia Department of Health North Metropolitan Health Service and received Ethics Approval in accordance with the Human Research Ethics Committee Protocol for Quality Activities. Consent for study participation was obtained verbally. In preparation for the study, the observation team was carefully selected to include a medical practitioner in psychiatry who was familiar with the working environment and experienced in the sensitive assessment of patients presenting with suicidal ideation in the emergency setting. A consultation process took place between the research team and emergency physicians in the department. An understanding was reached whereby any incident of concern that was uncovered during data collection would be conveyed to the departmental leadership in real time.

### Data collection

Data were collected at Sir Charles Gairdner Hospital, which is a tertiary hospital in the city of Perth, Western Australia. A team of 2 observers worked simultaneously to record instances of clinical interactions for patients presenting with risk of suicide or self-harm. The observation team comprised a Psychiatry Registrar and a postdoctoral research scientist. Potential participants were assessed for inclusion in the study upon presentation to the ED triage when clerked into the Emergency Department Information System (EDIS). Patients were considered suitable for inclusion if suicidal ideation or self-harm was listed in EDIS as a presenting complaint. Patients were consented for participation in this study prospectively as they met criteria for inclusion. Consent was sought at the earliest possible opportunity during which no active clinical interaction or assessment was taking place. Participant information approved by the Research Ethics Committee Chair was conferred to the participants by way of a discussion, and their verbal consent was obtained prior to each patient trajectory being included. Participants were informed that no identifying data about themselves, their condition or their treatment would be recorded as part of the study design. Participants were made aware that participation in the study, or refusal thereof would have no bearing on the clinical care they would receive and that they could revoke this consent at any time. Participants who were intoxicated on entering the ED were retrospectively consented once no longer intoxicated.

Observers collected data for each study participant by recording instances of clinical interactions along the patient’s trajectory through the ED. This included the mode of presentation, date and time of each interaction, the agents involved in each interaction (e.g., patient, associates of the patient, clinical staff, support and community health services, or emergency services), patient records or information systems accessed during the interaction, and actions resulting from an interaction such as referrals or discharge. Data were recorded in an event log developed specifically for this study (Table 1). The team of observers combined and cross-checked observations in the event log regularly during data collection to reduce the likelihood of data errors.

**Table 1:** Example event log: Data were collected in the form of an event log as illustrated by this table. Shown here are partial trajectories for 2 patients. Each row corresponds to an observed interaction, listed in chronological order. For each patient, we recorded the method of presentation. For each subsequent interaction, we recorded the date and time of the interaction, the agents involved (i.e., Patient, Triage Nurse, Consultant), and any information systems accessed during the interaction (i.e., reading from or writing to the Patient File, accessing digital patient record databases, completing risk assessment forms). We also recorded actions that took place as a result of interactions (i.e., referrals or discharge).

Patient	Presentation	Date	Time (24 h)	Agents	Information Systems	Action
1	Associate	01/MM/YYYY	12:52	Triage Nurse; Patient; Associate;	Emergency Department Information System (EDIS)	Referral to Emergency Medical
1	Associate	01/MM/YYYY	13:36	Consultant; Patient; Associate;	Patient File	Referral to Emergency Psychiatry
1	Associate	01/MM/YYYY	13:37	Consultant; Psychiatric Liaison Nurse;		
2	Ambulance	02/MM/YYYY	12:15	Triage Nurse; Paramedic;		
2	Ambulance	02/MM/YYYY	12:16	Triage Nurse; Patient;	EDIS	Referral to Emergency Medical
2	Ambulance	02/MM/YYYY	12:20	Triage Nurse; Paramedic;	EDIS	
2	Ambulance	02/MM/YYYY	12:26	Nurse; Paramedic;	Patient File	

Efforts were made to protect patient confidentiality as far as possible. The data collected were strictly nonclinical information only. Date information in the data was altered to minimize the possibility of retrospective data linkage. Interactions between patients and physicians were observed from a sufficient distance such that clinical conversations could not be heard. The researchers were never present in a confined environment that would have compromised physician–patient privilege. There was minimal impact on routine clinical processes. In instances where it was not clear if an interaction pertained to a study participant (e.g., a phone call or discussion between clinical staff), or involved access of one or more information systems (e.g., use of a computer terminal), clinical staff were approached at an appropriate time and briefly interviewed to establish the relevant details.

Observations were taken for a total of 101 h in the ED over a period of approximately 1 month spanning July and August in the year 2018, including 40 h during day shifts, 40 h during night shifts, and 21 additional hours of observations during day shifts specifically focused around the ED Psychiatry team. Observation hours were distributed between weekdays and weekends and were generally undertaken in 8-h shifts. A total of *n* = 213 interactions were observed from *n* = 36 patient trajectories in the ED.

Further observations were undertaken at the Sir Charles Gairdner Hospital Mental Health Observational Area (MHOA). The MHOA is an 8-bed short-stay psychiatric unit located physically adjacent to the ED and staffed by a multidisciplinary team. Its model of care emphasizes acute presentations that are expected to resolve within a 24- to 72-h duration of admission, or for patients whose disposition to a traditional acute psychiatric unit can be confirmed during that time. The MHOA receives patients from the ED, its sole admission pathway. ED Psychiatry and MHOA staff operate under the same clinical governance for all mental health staff on the campus. The MHOA was observed for a total of 17.5 h over 5 weekdays. A total of *n* = 59 interactions were observed from *n* = 7 patient trajectories in the MHOA.

The study population was reflective of a naturalistic sample of patients presenting to a tertiary ED in a metropolitan Australian city. No demographic details were captured in keeping with the study design. We refer readers to a study by Dragovic and colleagues [29] for baseline population characteristics of mental health presentations to tertiary hospitals in the same district operated by the Western Australia Department of Health North Metropolitan Health Service for the period of 25 January to 1 May 2019 (within 1 year after data collection for the present study).

### Data analysis and software

Data analysis were performed in Python using the packages Numpy [30], Pandas [31], NetworkX [32], Scikit-Learn [33], and Imbalanced-Learn [34]. Figures and data visualizations were prepared using Matplotlib [35] and NetworkX.

### Patient trajectory network and basic trajectory statistics

To map possible trajectories from the point of presentation to referral or discharge, we constructed a patient trajectory network using only the observational data. We defined nodes in the network representation of patient trajectories as the clinical team treating the patient, modes of presentation, and modes of discharge. An unweighted and directed edge was assigned between a pair nodes if we observed at least 1 instance of that transition in our data (i.e., a patient being moved from the ED to the Observation Ward). The combined data from all ED and MHOA observations were used to construct the patient trajectory network.

When computing the presentation type, trajectory time, interactions, and clinical staff types, we excluded patients that were observed only in the MHOA. We excluded this data because the MHOA served a different function than the other areas of the ED, providing a specialized short-stay environment for observation of at-risk mental health patients. This exclusion was also applied in the subsequent analysis of the clinical interaction network.

### Clinical interaction network

To investigate the flow of clinical information in the ED, we constructed a network model of the interactions between agents (i.e., the patient, doctors, or nurses) and clinical information systems observed along the combined set of patient trajectories. Minimal assumptions were imposed only to ensure that nonrealistic interactions were not erroneously included in the network (e.g., a hard-copy Patient File cannot directly interact with a digital records database). The network model was otherwise constructed entirely from the observational data. We then estimated the importance of individual agents and the overall efficiency of the system with respect to information flow based on quantitative measures of connectivity patterns in the network model, detailed as follows.

We define the clinical interaction network as comprising a set of nodes $V=\{i{\}}_{i=1}^{n}$, where each node *i* corresponds to one of the |V|=n total possible agents or information systems. The network is represented by an *n* by *n* adjacency matrix *A*. Elements of *A* are given by *a*_*i*,*j*_∈{0,1}, where *a*_*i*,*j*_ = 1 implies an unweighted bidirectional edge between nodes *i* and *j*. An edge was assigned between nodes *i* and *j* if and only if an interaction between the corresponding pair of agents or information systems occurred at least once in the combined data from all patients. The network edges can therefore be interpreted as communication channels for clinical information that would inform patient diagnosis and treatment. The operational policy of a hospital imparts intrinsic structure in the network that is not directly reflected in the event log data. For example, a recorded interaction may have involved the Patient, a Nurse, and the Patient File (which the Nurse may have read or appended information to). However, a Patient File is never accessed directly by the Patient. Therefore, edges between nodes corresponding to the Patient and the Patient File are considered forbidden and are excluded from the clinical interaction network by definition. In this study, forbidden edges included those (a) between clinical information systems; (b) between clinical information systems and agents that were not clinical staff at the hospital; (c) between the Psychiatric Services Online Information System (PSOLIS) or ED Psychiatry Handover Document (EDYHO), which are clinical information systems specific to psychiatry, and any agents that were not part of the Emergency Psychiatry team. We rendered the visualization of the clinical interaction network using the *spring_layout* function from NetworkX [32] that produces a force-directed graph layout.

To detect community structure in the network, we applied a greedy modularity maximization algorithm [36]. To estimate the importance of agents in the network, node centrality measures were computed based on the definitions by Newman [37], as briefly summarized here in Eqs (1) to (3). The degree centrality of a node is the number of edges connected to that node. The degree centrality of node *i* was computed as follows:

$$k_{i}=\sum_{j}a_{i,j}\;\mathrm{for}\;i,j\in V.$$ (1)

Closeness centrality is the inverse of the average distance from a given node to all other nodes in the network. The closeness centrality of node *i* was computed as follows:

$$c_{i}=\frac{\left(n-1\right)}{\sum_{j}d_{i,j}}\;\mathrm{for}\;i,j\in V:i\neq j,$$ (2)

where *d*_*i*,*j*_ is the length of the shortest path on the network between nodes *i* and *j*. The betweenness centrality of a node measures how often that node forms part of a path between other pairs of nodes. The clinical interaction network models the flow of information between agents. In this context, high betweenness would suggest that a node is important for passing information between other agents or different communities in the network. If a node with high betweenness becomes compromised this is likely to adversely impact the flow of information through the network more than for a node with low betweenness. The betweenness centrality of node *i* was computed as follows:

$$b_{i}=\sum_{j,k}\frac{\sigma (j,k|i)}{\sigma (j,k)}\;\mathrm{for}\;i,j,k\in V:i\neq j\;\mathrm{and}\;i\neq k,$$ (3)

where *σ*(*j*,*k*|*i*) equals the number of shortest paths between nodes *j* and *k* which pass through node *i*, and *σ*(*j*,*k*) equals the total number of shortest paths between nodes *j* and *k*. This particular variant of betweenness centrality is described in [38]. We further normalized *b*_*i*_ by the total number of possible paths through node *i* [39].

### Network vulnerability analysis

We applied a random shuffle algorithm to assess the degree to which the structure in our observed network was due to inherent structure in the data rather than randomness. The principle is that we generated an ensemble of networks which appeared similar to our clinical interaction network (they have the same number of nodes, node degrees, etc.) but were otherwise random. We then sought to answer the question of whether the observed clinical interaction network was different from random—and if so, how?

Specifically, random shuffling of the clinical interaction network was performed using a connected double-edge swap algorithm [40] to preserve local and global degree structure. This algorithm begins by randomly selecting 2 pairs of nodes (*i*,*j*) and (*v*,*u*) such that the nodes within each pair are connected (i.e., *a*_(*i*,*j*)_ = *a*_(*v*,*u*)_ = 1). The edges are then swapped so that the network has 2 new connected node pairs (*i*,*v*) and (*j*,*u*). This swap is only performed if: (a) the edges for new node pairs (*i*,*v*) and (*j*,*u*) did not already exist in the network; and (b) the network remains connected after the swap. If these conditions are not met, the edges for these 2 node pairs are left unchanged and the algorithm proceeds to attempt a swap with a different randomly selected set of node pairs. For this study, we imposed a further constraint that edges could only be swapped if the resulting network remained free of forbidden edges as defined for the clinical interaction network (henceforth referred to as the constrained connected double-edge swap algorithm). We generated 1,000 shuffled networks from independent sequences of random edge swaps to assess the likelihood of the observed network configuration. For each shuffle, we attempted 20,000 connected double-edge swaps, of which approximately 1,900 swaps were successful on average.

We assessed network vulnerability based on changes in closeness centrality and global efficiency [41] when a potentially vulnerable node was removed. Global efficiency measures how efficiently information propagates on a network. Assuming that efficiency of information flow between a pair of nodes *i* and *j* is inversely proportional to the shortest path between them *d*_*i*,*j*_, the global efficiency is the average over all node pairs, computed as follows:

$$g=\frac{1}{n(n-1)}\sum_{i,j}\frac{1}{d_{i,j}}\;\mathrm{for}\;i,j\in V:i\neq j.$$ (4)

To investigate strategies to mitigate against the adverse effects of a compromised node, we developed a simple greedy algorithm for the targeted addition of edges to increase global efficiency, as follows: (a) remove the compromised node from the network; (b) for each edge in a set of candidate edges, add the edge to the network which maximizes the increase in global efficiency; (c) repeat the previous step until all edges from the candidate set are added to the network. If a tie is encountered with respect to the increase in global efficiency, then this greedy algorithm is no longer guaranteed to find an optimal sequence for the addition of edges. Therefore, once a tie occurred we tested all possible permutations for the sequence of the remaining edges that had not yet been added. This allowed us to enumerate the complete set of optimal solutions. For our data and the specific set of candidate edges investigated in this study, there was a subset of edges that were tied with respect to their contributions to global efficiency, regardless of the order in which they were added to the network.

### Machine learning for predicting clinical referrals

To assess which agents and interactions were likely to precipitate a referral to the Emergency Psychiatry team, we trained a machine learning model to predict the referral point based on the evolving state of clinical interaction networks along individual patient trajectories. We call this the referral prediction model. The model was investigated to identify the features of the network (i.e., nodes and edges) that were most predictive of referral. We used machine learning for this task as a way of extracting structural information from the underlying data, independent of our own application driven bias. The machine learning algorithm was agnostic to our knowledge of the system and was simply applied to extract significant structural patterns from the data.

The referral prediction model was trained on data from trajectories for *n* = 20 patients who were referred from the Emergency Medical team to the Emergency Psychiatry team during the period of observation. A dynamic clinical interaction network *A*_*p*,*t*_ was iteratively constructed along the trajectory for each patient *p* and observation number *t* starting with an empty network *A*_*p*,0_. For each interaction along the trajectory, we added nodes and edges for the corresponding agents if they did not already exist in the network. This process continued up to and including the interaction which precipitated referral. The final state of *A*_*p*,*t*_ along the trajectory was assigned a positive class label *y*_*p*,*t*_ = 1, delineating the referral point. All other states of *A*_*p*,*t*_ were assigned the class label *y*_*p*,*t*_ = 0. Each state of *A*_*p*,*t*_ was mapped to a 1-dimensional binary feature vector:

$$X_{p,t}=[v_{1},v_{2},v_{3},\ldots ,v_{n}|a_{1,1},a_{1,2},\ldots a_{1,n},a_{2,1},a_{2,2},\ldots ,a_{n,n}],$$ (5)

where *v*_*i*_ is a Boolean variable representing the existence of node *i* in the network at time *t* for patient *p*, and *a*_*i*,*j*_ is the Boolean variable representing the existence of an edge between nodes *i* and *j*. The indices *i* and *j* correspond to those for the complete interaction network *A*.

The features *X*_*p*,*t*_ and labels *y*_*p*,*t*_ were then used to train a Bernoulli naive Bayes classifier with Laplace smoothing [42] to predict the referral point based on the dynamic network state. This machine learning algorithm was selected for the referral prediction model because it takes binary feature vectors as input, which matched the structure of our dynamic network state vector *X*_*p*,*t*_, and has an intuitive probabilistic interpretation. Briefly, this machine learning algorithm estimates the probability *P*(*y*|*X*) for class *y* and multivariate input feature vector *X*, under the assumption that each input feature is independent for a given class label. For example, in the context of our application the model assumes that the involvement of one agent or interaction in a patient trajectory occurred independently of any other. This simplifying assumption makes the estimation of *P*(*y*|*X*) more tractable in the case of high-dimensional data and a limited sample size, which would otherwise require a very large sample of observations to accurately estimate probabilities for the vast number of possible input vectors (up to $2^{\frac{n(n-1)}{2}+n}$ different possibilities for our dynamic network state vector *X*_*p*,*t*_). For these reasons, the Bernoulli naive Bayes classifier is well-suited for the psychiatric patient trajectory data gathered in this study, which are intrinsically high-dimensional and highly variable.

We hypothesized that some features of *X*_*p*,*t*_ would be more predictive than others and that it might be informative to identify the agents or interactions corresponding to these features. To investigate this, we used permutation feature importance [43] that quantifies the contribution of each feature in the model by measuring the change in a scoring metric when the data for that feature are randomly permuted. We used balanced accuracy [33] as the scoring metric because class labels are highly imbalanced—positive class labels (referral) typically only occur once in a patient trajectory through the ED and only account for 23% of the data (20 out of *n* = 87 interactions in *X*_*p*,*t*_ for our data). The balanced accuracy scoring metric is given by:

$$score=\frac{1}{2}\left(\frac{TP}{TP+FN}+\frac{TN}{TN+FP}\right),$$ (6)

where TP, FP, TN, and FN are the number of true positives, false positives, true negatives, and false negatives in the test data, respectively. We estimated the permutation feature importance for 10,000 randomly re-sampled 80:20 train/test splits of the data. Data were grouped such that observations from any given patient trajectory could not be split between the training and test sets. To avoid bias in the model due to highly imbalanced class labels, we used random over-sampling of the minority class to balance the data in each training split [34].

### Reporting

This study is reported as per the Strengthening the Reporting of Observational Studies in Epidemiology (STROBE) guideline (S1 STROBE Checklist).

## Results

### Characterizing patient trajectories as sequences of clinical interactions

**Fig 1:**
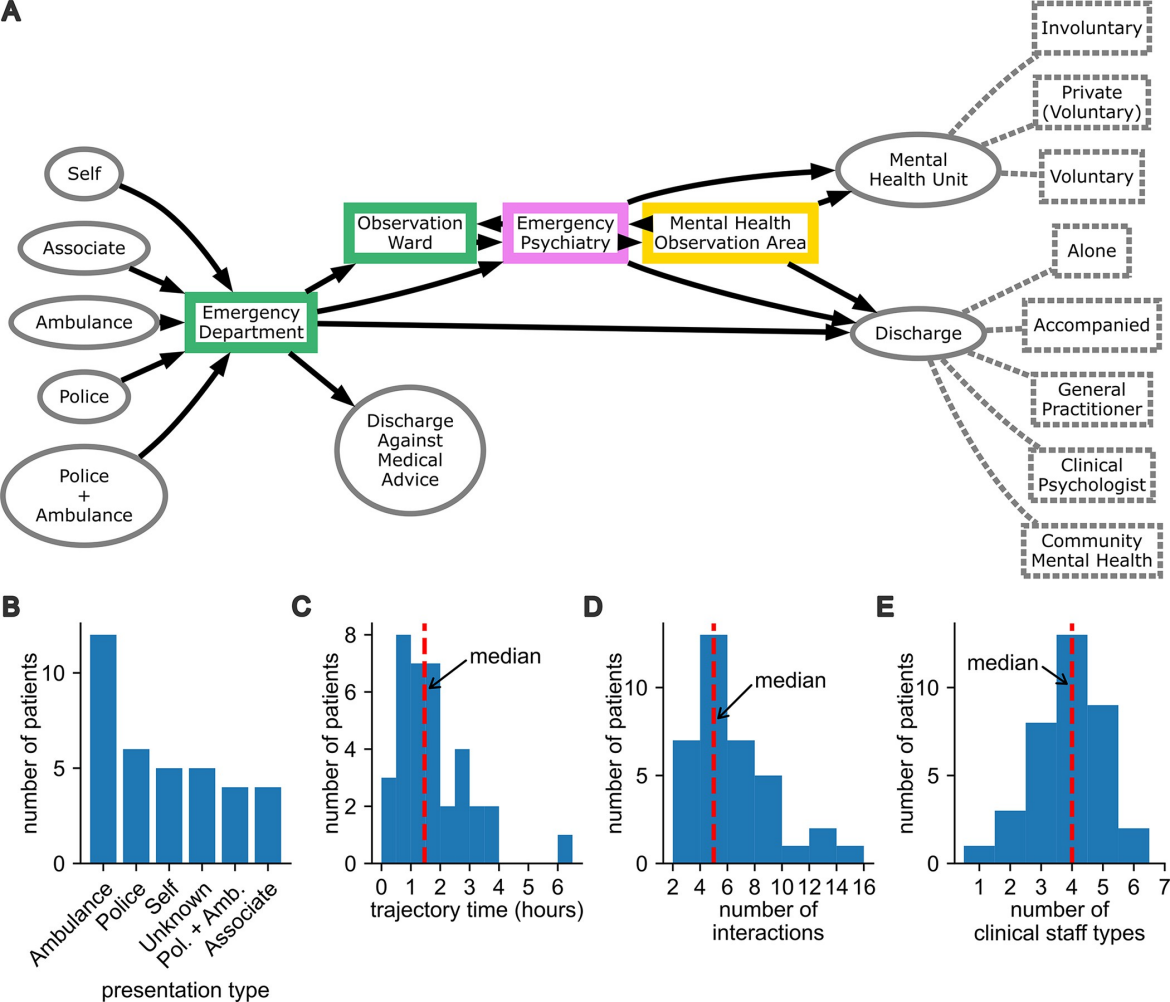
Mapping trajectories through an ED for patients at risk of suicide or self-harm. (A) A network representation of possible patient trajectories constructed from observations from a total of *n* = 43 patients. There were *n* = 36 observed within the ED (including the Observation Ward and Emergency Psychiatry) and *n* = 7 observed only within the MHOA. Solid arrows show the observed directional transitions from presentation through the different clinical teams to the point of discharge. The clinical team responsible for treating a patient at each point along the trajectory is represented by box color, where green is the Emergency Medical team, pink is the Emergency Psychiatry team, and yellow is the MHOA team. The nature of discharge or referral is indicated by the annotations with dashed gray lines. Histograms of (B) presentation type, (C) trajectory time which was the total time between the first and last observation in each patient trajectory, (D) the number of interactions observed per patient, and (E) the number of types of clinical staff involved per patient. Dashed red lines indicate the median of the data in each panel. ED, emergency department; MHOA, Mental Health Observational Area.

The patient trajectory network constructed from the observational data captured most of the transitions expected based on the operational structure of the ED (Fig 1A). This suggests that our data comprised a representative collection of possible patient trajectories. Discharge against medical advice was observed only once. This occurred while the patient was in the care of the Emergency Medical team, but such events were also possible at other points along a patient trajectory. The most common mode of presentation was by ambulance, police, or a combination of these (Fig 1B). The median trajectory time per patient was 1.5 h, 95% confidence interval (CI) [0.3, 3.8] (Fig 1C). Trajectory time was defined as the duration between the first and last observation made for each patient. Note that trajectory time is not equivalent to a patient’s length of stay because it was not always possible to observe complete trajectories based on our data collection protocol. Along each trajectory, we captured a median of 5 interactions (95% CI [2, 12]; Fig 1D) involving a median of 4 different types of clinical staff (95% CI [2, 5]; Fig 1E).

### A network model of interactions reflects clinical team structure and reveals agents important for information flow

**Fig 2:**
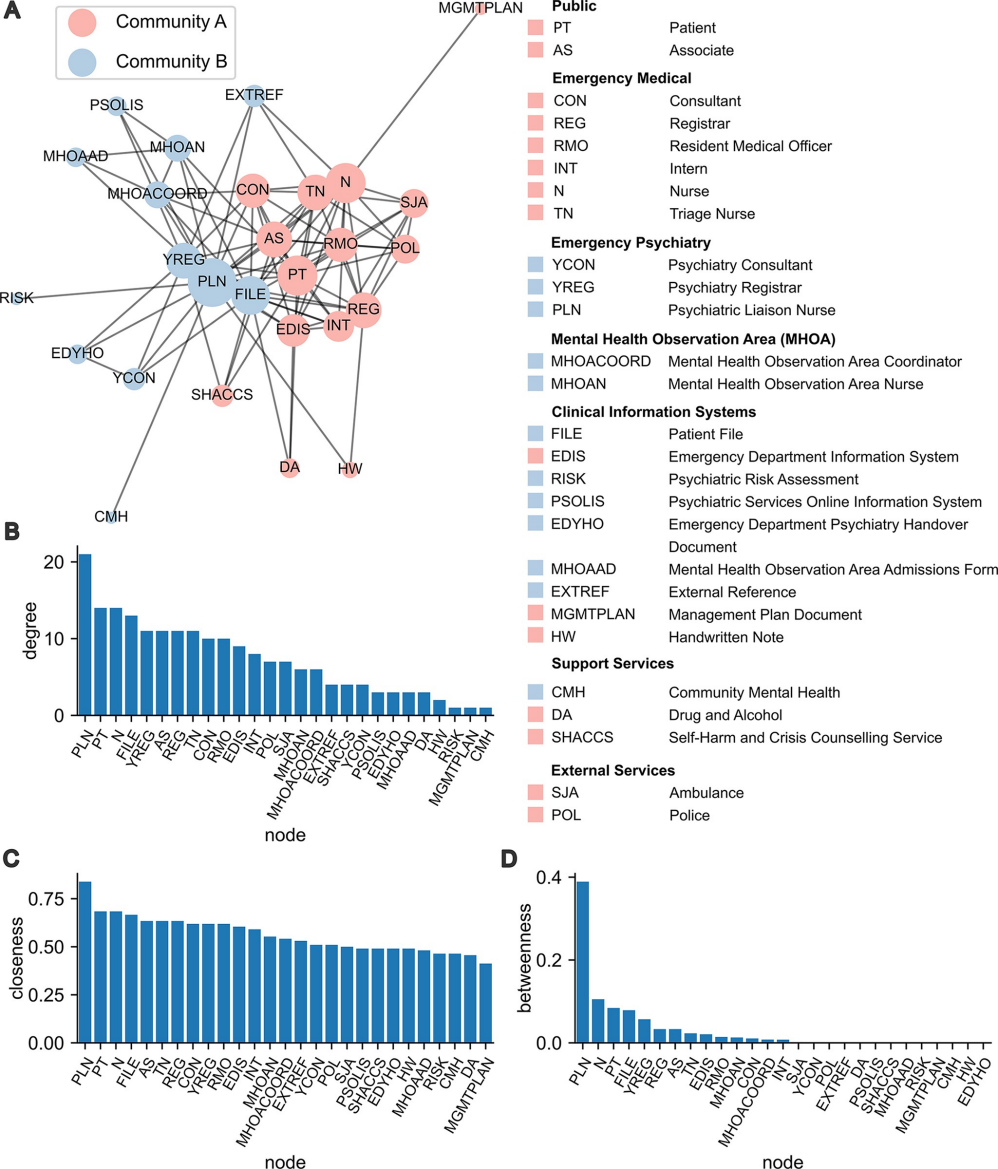
The clinical interaction network comprises 2 communities divided between the Emergency Medical and Emergency Psychiatry teams and their respective clinical information systems. (A) The interaction network of clinical staff, support services, external services, and information systems constructed from observations of *n* = 213 interactions for patient trajectories in the ED. Each node represents one of the listed agents. Node size reflects interaction frequency, where larger nodes represent agents that were involved in more interactions. Edges are unweighted and undirected, and represent the observation of at least 1 interaction between a pair of agents in the data. Two communities were identified by greedy modularity maximization. Community membership is indicated on both the network visualization and the glossary of agents. The network layout was generated using a force-directed graph algorithm. (B) Degree centrality, (C) closeness centrality, and (D) betweenness centrality of all nodes in the network show high importance of the PLN. ED, emergency department; PLN, Psychiatric Liaison Nurse.

The clinical interaction network constructed from observations recorded in the ED is shown in Fig 2A. Applying community detection to the network revealed a division between the Emergency Medical team and Emergency Psychiatry team based only on the patterns of communications and interactions in the data. The respective internal reporting structures of these teams presumably contributed to this division. However, patient outcomes are nonetheless likely to be dependent on effective and reliable communication between teams.

Therefore, we next computed measures of node centrality to quantify the importance of individual agents in the clinical interaction network with respect to information flow. Node degree quantifies the direct connectedness and activeness of a node. The Psychiatric Liaison Nurse (PLN), Patient, Nurse, and Patient File were most central in the clinical interaction network by node degree (Fig 2B). This result is unsurprising for the Patient, Patient File, and the Nurse given that the Patient is the focus of the interactions, the Patient File is the primary clinical record, and Nurses performed regular observations of the Patient. However, it was not immediately apparent as to why the PLN had the highest node degree. Closeness centrality quantifies how central a given node is within the overall structure of the network. The PLN also had the highest closeness centrality (Fig 2C). Values of this measure for all other nodes were relatively consistent. Betweenness centrality quantifies how essential a given node is for transport of information across the network. By this metric, the PLN again had the highest centrality, with a value more than 4 times greater than the agent with the next highest betweenness (Fig 2D). Overall, these results suggest that the PLN is a highly active and connected agent in the ED and may play a crucial role in communicating clinical information between other agents and treating teams.

### Assessing network vulnerabilities and reducing potential impacts via targeted algorithmic addition of edges

**Fig 3:**
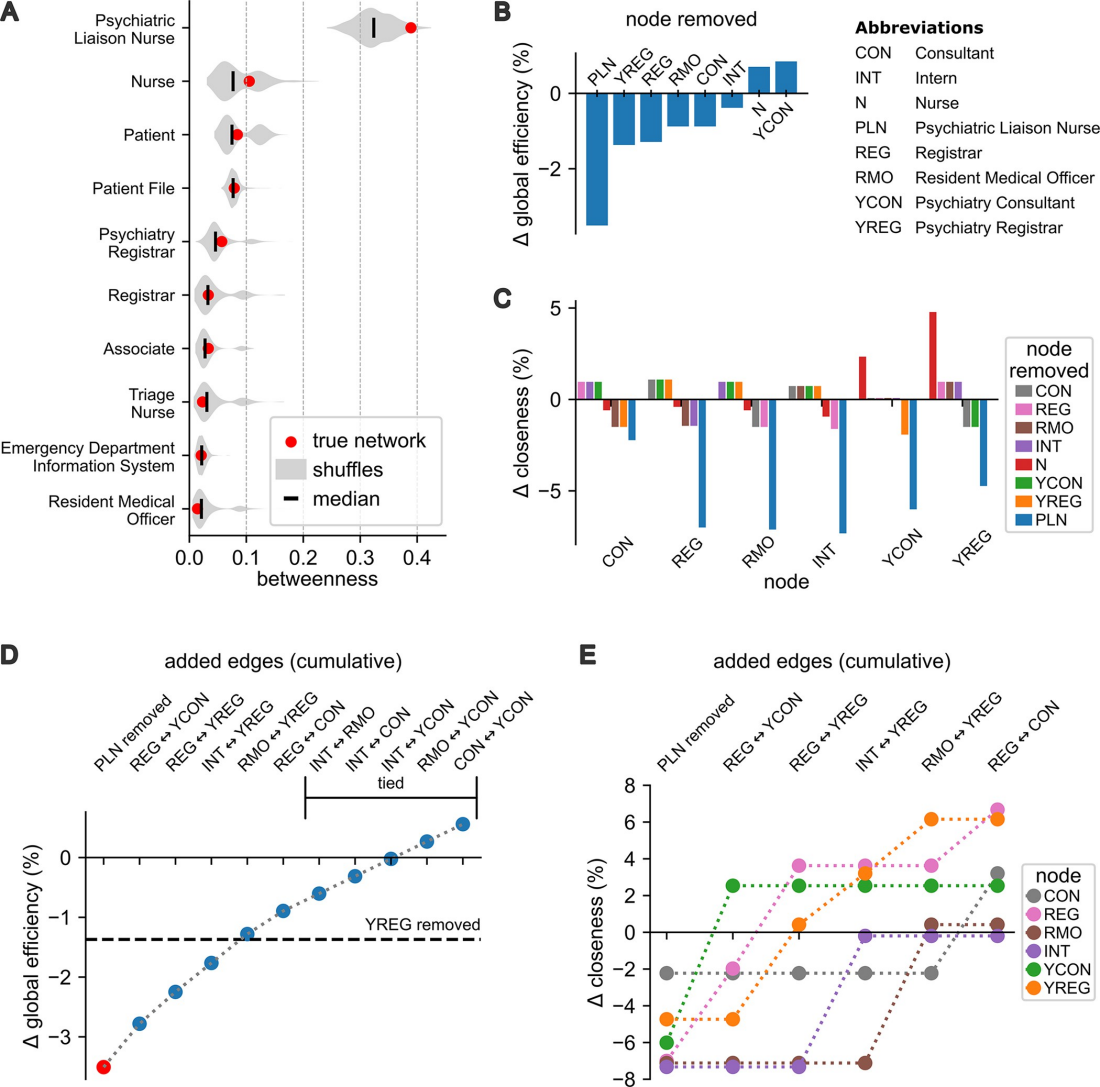
Network vulnerability to compromised function of the PLN is reduced by strengthening links between Emergency Medical and Emergency Psychiatry doctors. (A) The distributions of betweenness centrality shown as violin plots for 1,000 random shuffles of the clinical interaction network for the 10 nodes with the highest values for this statistic. The betweenness for the PLN in the true network was greater than the 95th percentile of the shuffles, indicating that this network may be more vulnerable to compromised function of the PLN than expected by chance in similar networks. (B) The change in global efficiency when key clinical staff were removed from the network. (C) The change in closeness centrality for doctors when key clinical staff were removed from the network. (D) Greedy cumulative addition of edges after the removal of the PLN shows that global efficiency could be restored to a level comparable to the removal of other key clinical staff (dashed line) by the addition of 4 edges between doctors from the Emergency Medical and Emergency Psychiatry teams. Edges marked as tied contributed equally to the increase in global efficiency regardless of order. (E) Closeness centrality for doctors in the network could be restored following the removal of the PLN by the same greedy addition of edges as in panel D. PLN, Psychiatric Liaison Nurse.

The high node degree and betweenness centrality of the PLN indicated a potential network vulnerability. If the function of the PLN was compromised this might adversely impact the communication of important information between clinical staff. We first sought to establish whether the high betweenness of the PLN resulted from the specific configuration of the clinical interaction network. The alternative hypothesis was that comparable values would arise by chance in similar networks that were configured randomly. To investigate, we generated randomly shuffled versions of the clinical interaction network using the constrained connected double-edge swap algorithm. Betweenness centrality for the PLN was significantly higher in the true network than for random shuffles (>95th percentile; Fig 3A). This was not the case for any other of the 10 most central agents by betweenness, which implies that the specific configuration of the clinical interaction network may have imposed an unexpectedly high load on the PLN with respect to information transfer. Furthermore, the shuffling algorithm we applied explicitly preserves the degree of each node when shuffling. Therefore, this result also ruled out the possibility that the PLN had unexpectedly high betweenness only because it was highly connected, indicating that the position of the agent in the network is important as well as connectivity.

Next, we investigated how information flow might be impacted if the function of the PLN was compromised. Global efficiency measures how efficiently information flows between all pairs of nodes averaged over the network. When the PLN was removed from the network model, global efficiency dropped by more than 3.5% (Fig 3B). This was more than 2.5 times the impact of removing the agent with the next largest impact. The closeness centrality of doctors was reduced considerably more by the removal of the PLN from the network compared with the removal of other clinical staff (Fig 3C). Together, these results indicate that the structure of clinical interactions in the ED may make the system especially vulnerable to compromised function of the PLN. This vulnerability presents a risk to the flow of clinical information between agents responsible for decision-making along patient trajectories.

We sought a strategy to mitigate this risk by targeted addition of edges on the clinical interaction network between doctors in the Emergency Medical and Emergency Psychiatry teams. We began by removing the PLN from the network then used a greedy algorithm to add edges one at a time to maximize the increase in global efficiency. The 4 edges which contributed most to restoring global efficiency were edges that linked a doctor from the Emergency Medical team to one from the Emergency Psychiatry team (Fig 3D). The addition of these 4 edges restored global efficiency to a level comparable to the loss of efficiency that would arise from the compromised function of other clinical staff (Fig 3B). Furthermore, closeness centrality for doctors was fully restored with the exception of the Intern who required one more edge (Fig 3E). In summary, these results imply that the network vulnerability caused by the high centrality of the PLN could be reduced by increasing communication between doctors from the Emergency Medical and Emergency Psychiatry teams.

### Identifying types of interactions that precipitate clinical handovers with machine learning on clinical interaction networks

**Fig 4:**
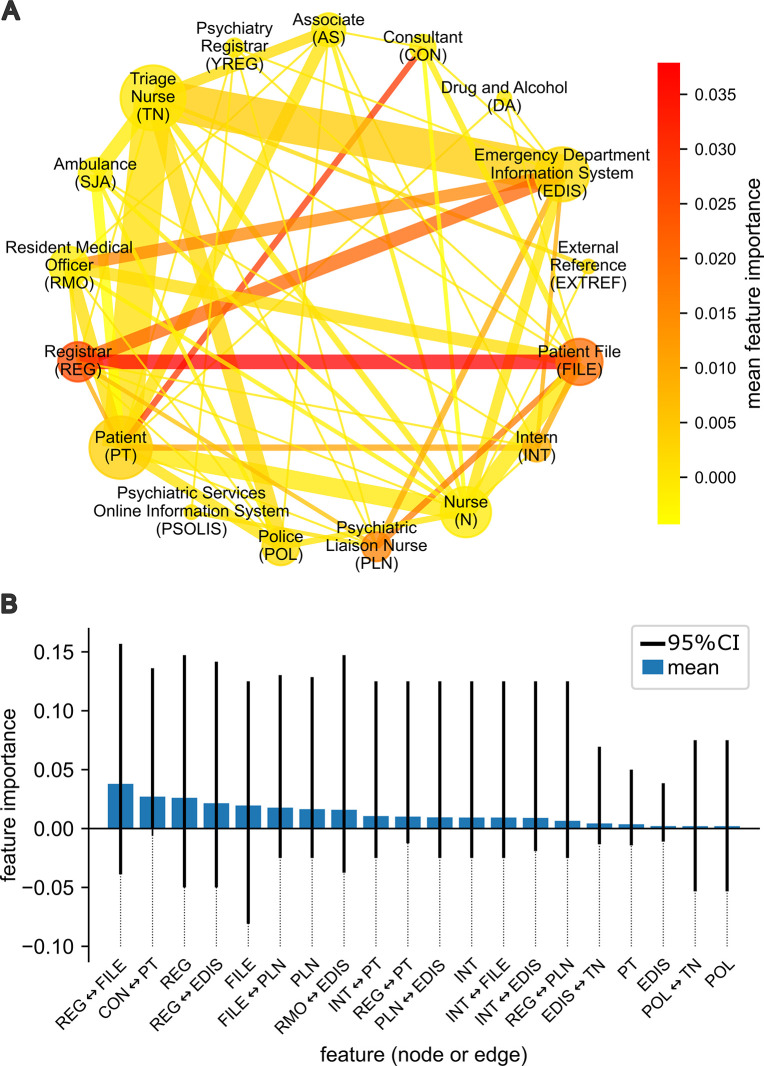
Machine learning on network features reveals agents and interactions that predict the referral from the Emergency Medical team to Emergency Psychiatry. (A) A circular visualization of the average clinical interaction network for patient trajectories where there was a clinical referral to the Emergency Psychiatry team, averaged up to the point of the referral (*n* = 20 patients, *n* = 87 interactions). Node sizes and edge weights reflect the respective relative number of instances for which agents and interactions were observed in this subset of trajectories, where larger size or heavier weight indicates more instances of observation. The color of a node or edge shows its importance for predicting the clinical referral in a subsequent interaction, as computed by permutation feature importance using a Bernoulli naive Bayes classifier trained on the set of nodes and edges at each step of a patient trajectory. Nodes are arranged in the circle based on alphabetical ordering of their respective abbreviations in a clockwise direction. (B) Mean feature importance and 95% CI for the top 20 nodes or edges over 10,000 randomly re-sampled 80:20 train/test splits of the data. CI, confidence interval.

We then studied how patterns of interactions influenced clinical decision points along patient trajectories. Specifically, we used our referral prediction model to quantify the relative importance of different features from the clinical interaction network for predicting the point of referral to Emergency Psychiatry. The mean balanced accuracy of the model averaged over all randomly re-sampled training and testing splits of the data was 82%, (95% CI [59%, 100%]; model performance was measured on the test data). We found that an interaction between a Registrar and the Patient File, or between a Consultant and the Patient were most predictive of referral on average (Fig 4A and 4B). The next most predictive events were the involvement of a Registrar in the patient trajectory, an interaction between a Registrar and EDIS, and any access of the Patient File. The predictive power of all network features had high variance (Fig 4B). For example, the Patient File often had a negative value for permutation importance implying that the contribution of this feature to the model could be worse than random. High variance in feature importance was likely a result of the high dimensionality of the feature space relative to the number of observations, coupled with the often complex nature of patient trajectories through the ED. However, the model suggests that the involvement of senior doctors in a patient’s trajectory (i.e., a Consultant or Registrar) was more likely to precipitate referral to ED Psychiatry than the involvement of junior doctors (i.e., a Resident Medical Officer or Intern).

## Discussion

This study has introduced a quantitative research framework, new to the best of our knowledge, for investigating the provision of psychiatric care in emergency healthcare settings with a focus on patients with suicide or self-harm risk. By embedding observers in a tertiary hospital ED, we collected data to construct network models of patient trajectories and clinical interactions, respectively. The clinical interaction network had a community structure reflecting the operational division between medical and psychiatric teams. This model indicated that the PLN likely played a crucial role in gathering and communicating clinical information between teams, carrying a considerably higher load than other clinicians based on measures of network centrality. Further analysis suggested that this unexpectedly high load may create a risk whereby compromised function of the PLN could lead to reduced information flow between clinicians that negatively impacts patient care. We then used a targeted algorithmic approach to show that this risk might be mitigated by increasing communication between doctors in the Emergency Medical and Emergency Psychiatry teams. Finally, we used a machine learning model trained on dynamic network features to identify which clinical interactions were most likely to result in a psychiatric referral.

The unexpectedly high importance of the PLN revealed by our quantitative analysis has implications for operational models that incorporate this or similar clinical roles. PLNs are generally recognized as being beneficial for the provision of mental health care in EDs with studies often citing merits such as reduced wait times, positive patient experience, and therapeutic benefits [9,17,18,22,24,44–50]. Notably, qualitative studies have described how PLNs have an important function in communicating information and coordinating patient care including providing assessments and recommendations to doctors, and serving as a link to other hospital services (e.g., alcohol and other drug services) and community mental health services [18,24]. This agrees with our observation that the PLN had high centrality in the clinical interaction network. These same studies also reported instances of staff becoming reliant on PLNs and facing considerable impact on workload in their absence [18] and that PLNs can feel unsupported and unsafe due to feeling overloaded with responsibility [24]. These reports are congruent with our finding that high load on the PLN within the clinical interaction network may pose a risk to the function of the system and patient outcomes. This shows that our approach appears to capture useful and interpretable information about the implementation of emergency psychiatric care. The advantage of our framework is that it allows for quantification and statistical comparison of different operational models and policies. However, the aforementioned qualitative studies and the present study were undertaken in different hospitals with different operation models. Therefore, future research should seek to validate the network model via a mixed-methods study encompassing both staff interviews and network analysis of clinical interactions in a range of different hospitals and healthcare settings.

We used the clinical interaction network to show that potential system vulnerabilities associated with the high centrality of the PLN might be mitigated by strengthening lines of communication between members of the Emergency Medical and Emergency Psychiatry teams. This echoes the more general and well-established finding of the importance of multidisciplinary collaboration and integration for the delivery of effective psychiatric care in emergency settings [24,26,44,47,51]. Furthermore, a recent study has also identified discrepancies between actual patterns of communication between clinical staff in practice compared with reporting structures as intended in the operational model based on a qualitative study [45]. The quantitative research framework we have developed facilitates direct assessment and comparison of actual patterns of communication against policy and organizational expectations in the evaluation of clinical practice. In addition, we have shown how machine learning classifiers can be used in conjunction with the clinical interaction network to understand how patterns of communication impact patient pathways and clinical decision points.

Recent reports highlight the need for new evidence-based measures to evaluate the implementation of clinical pathways in emergency psychiatry [11,20,23,28], and more data-driven methods for investigating the behavioral aspects of emergency care more broadly, where research is currently limited [52]. The data-driven research framework and quantitative metrics presented here have considerable potential for application and adaption to address a range of challenges in emergency psychiatry. For example, several studies have reported that mental health patients have negative experiences of emergency care due to issues including wait times, lack of appropriate spaces for the provision of care, and negative attitudes of staff during interactions [12,19,26,47]. By incorporating multimodal data collection of interaction observations, patient interviews, and the appropriate linkage of clinical records, our framework could be extended to evaluate the effectiveness of clinical pathways in terms of patient experience, patient flow, or patient outcomes such as referrals or re-admission rates. In addition, mental health patients who self-present can differ markedly from those brought in by ambulance or police, but details about how their subsequent clinical pathways through emergency care differ are not well understood [53]. Our network-based approach would be ideally suited for mapping, measuring, and comparing the nature of patient trajectories for different types of presentations to improve resource allocation, or to develop targeted clinical pathways to enhance treatment outcomes and efficiency for different patient groups.

Methodological approaches for process improvement, resource allocation, and management in healthcare traditionally employ simulation-based modeling [52], but an increasing number of studies are being conducted to explore the use of process mining to assess healthcare systems based on data from electronic records [54]. Similarly to our approach, process mining can be applied to map care pathways and assess process implementation as occurs in practice, which can deviate from policy, standards, or best-practice guidelines for reasons including patient complexity, clinical acumen, or organizational structure [55]. However, process mining and related methods are often limited by the availability, quality, and completeness of the data that can be extracted from electronic record databases [52,55]. In this context, our research framework serves as a complementary approach to process mining analysis and provides additional insights about clinical practice and information flow not captured in electronic records.

The limitations of our study include the sample size relative to the complexity of the system, which likely caused the high variance in our estimates of feature importance for predicting clinical decision points. Future studies utilizing our framework may benefit from a larger sample size. This would also enable the estimation of transition probabilities along patient pathways and the frequency of different interactions in the network model to provide a more accurate characterization of information flow. In addition, we restricted data collection to nonclinical information. This minimized the impact of our data collection protocol on normal clinical processes, but meant that we could not factor in quality or quantity of information transferred per interaction when modeling information flow. To maintain a minimally invasive data collection protocol and improve the inference of information flow in future research, it may be informative to record the duration of interactions and factor them into the construction of the clinical interaction network. The duration of an interaction may be related to the amount of information transferred between the agents involved. Deploying additional observers and retrospectively augmenting data using medical records would also reduce the risk of sampling bias during data collection.

In summary, our data-driven research framework for mapping patient trajectories in a tertiary hospital ED provides a quantitative approach for the assessment and improvement of operational models and clinical practice in the provision of emergency mental health care. We have shown that PLNs play an important role in communicating clinical information, but suggest that care should be taken when defining role responsibilities and managing such positions to avoid overloading staff. Furthermore, our modeling indicated that improving interdisciplinary communication in emergency psychiatry can make operational models more robust against vulnerabilities arising from high load on the PLN. To conclude, we note that while this study focused specifically on patients with suicide or self-harm risk, our framework could be applied equivalently to investigate other aspects of healthcare service delivery, including different medical specialties, other patient groups or demographics, or alternative settings such as community mental health clinics.

## Supporting information

S1 STROBE Checklist Strengthening the Reporting of Observational Studies in Epidemiology (STROBE) checklist. (DOCX)

## Abbreviations

CI: confidence interval
ED: emergency department
EDIS: Emergency Department Information System
MHOA: Mental Health Observational Area
PLN: Psychiatric Liaison Nurse
PSOLIS: Psychiatric Services Online Information System
