# Supplementary material for: Mapping clinical interactions in an Australian tertiary hospital emergency department for patients presenting with risk of suicide or self-harm: Network modeling from observational data
Source: PLoS Med. 2024 Jan 12;21(1):e1004241. doi: 10.1371/journal.pmed.1004241 (PMC10786386; doi:10.1371/journal.pmed.1004241)
Supplement: S1 STROBE Checklist — (DOCX) [file pmed.1004241.s001.docx]

STROBE Statement—checklist of items that should be included in reports of observational studies

|  | Item No | Recommendation | Location |
| --- | --- | --- | --- |
| **Title and abstract** | 1 | (*a*) Indicate the study’s design with a commonly used term in the title or the abstract | Title, Abstract |
|  |  | (*b*) Provide in the abstract an informative and balanced summary of what was done and what was found | Abstract |
| Introduction | | |  |
| Background/rationale | 2 | Explain the scientific background and rationale for the investigation being reported | Introduction 1-4 |
| Objectives | 3 | State specific objectives, including any prespecified hypotheses | Introduction 5 |
| Methods | | |  |
| Study design | 4 | Present key elements of study design early in the paper | Abstract  Methods – Data collection 2-7 |
| Setting | 5 | Describe the setting, locations, and relevant dates, including periods of recruitment, exposure, follow-up, and data collection | Methods – Ethics 1  Methods – Data collection 1-6 |
| Participants | 6 | (*a*) *Cohort study*—Give the eligibility criteria, and the sources and methods of selection of participants. Describe methods of follow-up  *Case-control study*—Give the eligibility criteria, and the sources and methods of case ascertainment and control selection. Give the rationale for the choice of cases and controls  *Cross-sectional study*—Give the eligibility criteria, and the sources and methods of selection of participants | Methods – Data collection 1 |
|  |  | (*b*) *Cohort study*—For matched studies, give matching criteria and number of exposed and unexposed  *Case-control study*—For matched studies, give matching criteria and the number of controls per case | N/A |
| Variables | 7 | Clearly define all outcomes, exposures, predictors, potential confounders, and effect modifiers. Give diagnostic criteria, if applicable | Methods – Data collection 2 |
| Data sources/ measurement | 8* | For each variable of interest, give sources of data and details of methods of assessment (measurement). Describe comparability of assessment methods if there is more than one group | Methods – Data collection 2 |
| Bias | 9 | Describe any efforts to address potential sources of bias | Methods – Data collection 2, 6 |
| Study size | 10 | Explain how the study size was arrived at | N/A |
| Quantitative variables | 11 | Explain how quantitative variables were handled in the analyses. If applicable, describe which groupings were chosen and why | Methods – Data analysis and software |
| Statistical methods | 12 | (*a*) Describe all statistical methods, including those used to control for confounding | Methods – Patient trajectory network and basic trajectory statistics (all paragraphs)  Methods – Clinical interaction network (all paragraphs)  Methods – Network vulnerability analysis (all paragraphs)  Methods – Machine learning for predicting clinical referrals (all paragraphs) |
|  |  | (*b*) Describe any methods used to examine subgroups and interactions | Methods – Clinical interaction network (all paragraphs)  Methods – Network vulnerability analysis (all paragraphs)  Methods – Machine learning for predicting clinical referrals (all paragraphs) |
|  |  | (*c*) Explain how missing data were addressed | N/A |
|  |  | (*d*) *Cohort study*—If applicable, explain how loss to follow-up was addressed  *Case-control study*—If applicable, explain how matching of cases and controls was addressed  *Cross-sectional study*—If applicable, describe analytical methods taking account of sampling strategy | N/A |
|  |  | (*e*) Describe any sensitivity analyses | Methods – Network vulnerability analysis (all paragraphs)  Methods – Machine learning for predicting clinical referrals 4 |

Continued on next page

| Results | | |  |
| --- | --- | --- | --- |
| Participants | 13* | (a) Report numbers of individuals at each stage of study—eg numbers potentially eligible, examined for eligibility, confirmed eligible, included in the study, completing follow-up, and analysed | Methods – Data collection 4-5  Methods - Machine learning for predicting clinical referrals 2 |
|  |  | (b) Give reasons for non-participation at each stage | Methods – Patient trajectory network and basic trajectory statistics 2  Methods - Machine learning for predicting clinical referrals 2 |
|  |  | (c) Consider use of a flow diagram | N/A |
| Descriptive data | 14* | (a) Give characteristics of study participants (eg demographic, clinical, social) and information on exposures and potential confounders | Methods – Data collection 6 |
|  |  | (b) Indicate number of participants with missing data for each variable of interest | N/A |
|  |  | (c) *Cohort study*—Summarise follow-up time (eg, average and total amount) | N/A |
| Outcome data | 15* | *Cohort study*—Report numbers of outcome events or summary measures over time | N/A |
|  |  | *Case-control study—*Report numbers in each exposure category, or summary measures of exposure | N/A |
|  |  | *Cross-sectional study—*Report numbers of outcome events or summary measures | Results – Characterising patient trajectories as sequences of clinical interactions 1 |
| Main results | 16 | (*a*) Give unadjusted estimates and, if applicable, confounder-adjusted estimates and their precision (eg, 95% confidence interval). Make clear which confounders were adjusted for and why they were included | Results – Characterising patient trajectories as sequences of clinical interactions 1  Results – A network model of interactions reflects clinical team structure and reveals agents important for information flow 1  Results – Assessing network vulnerabilities and reducing potential impacts via targeted algorithmic addition of edges 1-3  Results – Identifying types of interactions that precipitate clinical handovers with machine learning on interaction networks 1 |
|  |  | (*b*) Report category boundaries when continuous variables were categorized | N/A |
|  |  | (*c*) If relevant, consider translating estimates of relative risk into absolute risk for a meaningful time period | N/A |
| Other analyses | 17 | Report other analyses done—eg analyses of subgroups and interactions, and sensitivity analyses | N/A |
| Discussion | | |  |
| Key results | 18 | Summarise key results with reference to study objectives | Discussion 1 |
| Limitations | 19 | Discuss limitations of the study, taking into account sources of potential bias or imprecision. Discuss both direction and magnitude of any potential bias | Discussion 6 |
| Interpretation | 20 | Give a cautious overall interpretation of results considering objectives, limitations, multiplicity of analyses, results from similar studies, and other relevant evidence | Discussion 2-5 |
| Generalisability | 21 | Discuss the generalisability (external validity) of the study results | Discussion 7 |
| Other information | | |  |
| Funding | 22 | Give the source of funding and the role of the funders for the present study and, if applicable, for the original study on which the present article is based | Submission form |
